# T252. TREATMENT DELAY AND OUTCOME COMPARISON OF EXTENDED EARLY INTERVENTION SERVICE AND STANDARD PSYCHIATRIC CARE FOR ADULTS PRESENTING WITH FIRST-EPISODE PSYCHOSIS IN HONG KONG

**DOI:** 10.1093/schbul/sby016.528

**Published:** 2018-04-01

**Authors:** Wing Chung Chang, Chun Ho, Chi Fai Or, Tsz Ting Liu, Fu Chun Lau, On Ki Chu, Lai Ming Hui, Kit Wa Chan, Ho Ming Lee, Yi Nam Suen, Eric Chen

**Affiliations:** The University of Hong Kong

## Abstract

**Background:**

A territory-wide specialized early intervention (EI) service for psychosis (EASY) has been implemented in Hong Kong since 2001, providing 2-year phase-specific early assessment and clinical care to young people aged 15–25 years presenting with first-episode psychosis (FEP). Previous evaluation demonstrated superiority of EASY programme over standard care in outcome improvement in FEP. Recently, EASY has been extended to provide 3-year EI service to FEP patients aged 15 to 64 years. However, effectiveness of EI on adult FEP populations has not been well examined.

**Methods:**

This study adopted case versus historical-control design, comparing patients received 3-year EASY treatment (EI group) with those managed by standard psychiatric care (SC group) prior to implementation of EASY extension in terms of treatment delay and outcomes in symptom and functioning. In total, 320 Chinese adult FEP patients aged 26–55 years (160 in EI group, 160 in SC group) were included in the study. Retrospective record review detailing service utilization over 3-year treatment period was conducted. Follow-up interview assessment (on average 48.3 months after service entry) encompassing premorbid adjustment, duration of untreated psychosis (DUP), clinical (Positive and Negative Syndrome Scale, PANSS; Calgary Depression Scale, CDS), functional (Role Functioning Scale, RFS) and treatment profiles was administered. Comparison analyses on DUP and service utilization were based on record review data of 320 patients. Clinical and functional outcome analyses focused on data collected from follow-up interview assessment (251 patients completed follow-up assessment, 130 from EI and 121 from SC groups).

**Results:**

EI and SC groups were comparable regarding demographics, premorbid and baseline characteristics, except the use of second-generation antipsychotic (SGA) treatment (EI patients were more likely to receive SGA than SC patients). EI patients had significantly shorter DUP than SC counterparts (p=0.015). Regarding follow-up outcomes, EI patients displayed lower levels of negative (p=0.044) and depressive symptoms (p=0.055), higher scores in RFS immediate social network (p=0.027) and lower rates of service disengagement (p=0.048) than SC patients even when SGA use and DUP were adjusted as covariates in analysis of covariance for comparison. There were no significant group differences in admission and suicide rates.

**Discussion:**

Our results indicate that extended EASY service achieve favorable outcomes in adult FEP patients on shortening of treatment delay and improvement in negative symptoms and social functioning, and service disengagement reduction. Further evaluation is required to assess the sustainability of positive effects.

